# Chronic Cystitis—Perineal Cystotomy

**Published:** 1872-10

**Authors:** Robert Battey

**Affiliations:** Rome, Georgia


### CHRONIC CYSTITIS—PERINEAL CYSTOTOMY.

*To the Editor of the Medical Record.*

SIR.—In the 15th of July number of your journal, Dr. Charles K. Briddon remarks :

"Cystotomy, and by that I mean cutting into the bladder, was proposed some years ago by Prof. Willard Parker, as a remedy for those intractable cases of chronic cystitis that refuse to yield to other means. I am not aware that it has been done on the male. I do not think the wound could be kept open long enough for the bladder to recover itself without the assistance of a canula, which would not be tolerated in an inflamed organ."

It will be of interest to Dr. Briddon, and perhaps to others, to know that this operation was done by me, precisely as he states it, in May, 1869, and was briefly reported to the Georgia Medical Association. The report of the case may be found in the current June number of the Medical COMPANION, Atlanta, Ga. If Dr. Briddon will furnish me his address, I will, with pleasure, forward him the notes of the case.

In my case, the operation being, as I supposed, yet untried, was reserved for a last resort; and was done only when the patient seemed near his end. It was thought to be fully justifiable at the time as a palliative measure to relieve the sufferings of the patient, which had become continuous and very excruciating.

The relief afforded was immediate and most gratifying. Life was prolonged for eighteen months, and was for the most part comfortable and enjoyable. For a time, hopes were entertained that the proceeding would prove curative rather than palliative merely, as had been previously contemplated.

The inflamed bladder tolerated the "canula" (India-rubber tubing) admirably; indeed, so great was the relief afforded by it to the patient, he made little or no complaint of the presence of the tube or any irritation from it. The fistula left on its removal gave him an option either to evacuate the urine *per urethram* in the standing posture, or by the fistula, in a squatting position, at his pleasure; and this privilege he esteemed so highly that he would not contemplate the closure of the fistula under any circumstances. By voluntary muscular power he could control the escape of urine by the fistula.

So satisfied am I with the experience of the one case, I shall not hesitate to do the operation earlier when suitable opportunity offers, in the expectation that it may prove curative.

In conclusion, I may remark that I have found no local wash in these cases at all comparable to the solutions of chlorate potassa.

ROBERT BATTEY, M. D.

Rome, Georgia, August 6th, 1872.

